# Performance of deep learning models for automatic histopathological grading of meningiomas: a systematic review and meta-analysis

**DOI:** 10.3389/fneur.2025.1536751

**Published:** 2025-05-13

**Authors:** Parsia Noori Mirtaheri, Matin Akhbari, Farnaz Najafi, Hoda Mehrabi, Ali Babapour, Zahra Rahimian, Amirhossein Rigi, Saeid Rahbarbaghbani, Hesam Mobaraki, Sanaz Masoumi, Danial Nouri, Seyedeh-Tarlan Mirzohreh, Seyyed Kiarash Sadat Rafiei, Mahsa Asadi Anar, Zahra Golkar, Yasaman Asadollah Salmanpour, Ali Vesali Mahmoud, Mohammad Sadra Gholami Chahkand, Maryam Khodaei

**Affiliations:** ^1^School of Medicine, Iran University of Medical Sciences, Tehran, Iran; ^2^Department of Neurosurgery, Ege University Faculty of Medicine, Izmir, Türkiye; ^3^School of Medicine, Islamic Azad University of Medical Sciences, Tehran, Iran; ^4^Student Research Committee, School of Medicine, Arak University of Medical Sciences, Arak, Iran; ^5^Department of Computer Science, Tabari Institute of Higher Education, Tehran, Iran; ^6^School of Medicine, Shiraz University of Medical Sciences, Shiraz, Iran; ^7^Department of Radiology, Shahid Beheshti University of Medical Sciences, Tehran, Iran; ^8^Faculty of Medicine, Istanbul Yeni Yuzyil University, Istanbul, Türkiye; ^9^Yas Hospital Complex, Tehran University of Medical Sciences, Tehran, Iran; ^10^School of Medicine, Shahid Beheshti University of Medical Sciences, Tehran, Iran; ^11^Faculty of Medicine, Tabriz University of Medical Sciences, Tabriz, Iran; ^12^Student Research Committee, School of Medicine, Shahid Beheshti University of Medical Sciences, Tehran, Iran; ^13^College of Medicine, University of Arizona, Tucson, AZ, United States; ^14^Student Research Committee, Isfahan University of Medical Sciences, Isfahan, Iran; ^15^Student Research Committee, Islamic Azad University Science and Research Branch, Tehran, Iran; ^16^Department of Physiology, Buali Sina University, Hamedan, Iran; ^17^Student Research Committee, School of Medicine, Golestan University of Medical Sciences, Gorgan, Iran; ^18^Department of Clinical Biochemistry, School of Medicine, Shahid Beheshti University of Medical Sciences, Tehran, Iran

**Keywords:** histopathological grading, deep learning, meningioma, meta-analysis, AI

## Abstract

**Background:**

Accurate preoperative grading of meningiomas is crucial for selecting the most suitable treatment strategies and predicting patient outcomes. Traditional MRI-based assessments are often insufficient to distinguish between low- and high-grade meningiomas reliably. Deep learning (DL) models have emerged as promising tools for automated histopathological grading using imaging data. This systematic review and meta-analysis aimed to comprehensively evaluate the diagnostic performance of deep learning (DL) models for meningioma grading.

**Methods:**

This study was conducted in accordance with the PRISMA-DTA guidelines and was prospectively registered on the Open Science Framework. A systematic search of PubMed, Scopus, and Web of Science was performed up to March 2025. Studies using DL models to classify meningiomas based on imaging data were included. A random-effects meta-analysis was used to pool sensitivity, specificity, accuracy, and area under the receiver operating characteristic curve (AUC). A bivariate random-effects model was used to fit the summary receiver operating characteristic (SROC) curve. Study quality was assessed using the Newcastle-Ottawa Scale, and publication bias was evaluated using Egger's test.

**Results:**

Twenty-seven studies involving 13,130 patients were included. The pooled sensitivity was 92.31% (95% CI: 92.1–92.52%), specificity 95.3% (95% CI: 95.11–95.48%), and accuracy 97.97% (95% CI: 97.35–97.98%), with an AUC of 0.97 (95% CI: 0.96–0.98). The bivariate SROC curve demonstrated excellent diagnostic performance, characterized by a relatively narrow 95% confidence interval despite moderate to high heterogeneity (I^2^ = 79.7%, *p* < 0.001).

**Conclusion:**

DL models demonstrate high diagnostic accuracy for automatic meningioma grading and could serve as valuable clinical decision-support tools.

**Systematic review registration:**

DOI: 10.17605/OSF.IO/RXEBM

## Introduction

Meningioma is a particular sort of brain tumor that originates from arachnoid cap cells, and it is the most prevalent primary brain tumor (1, 2). This tumor is usually benign and is most commonly seen in older individuals, particularly females (2). Although it is a benign tumor because of its intracranial location, meningioma can result in severe consequences (3). Today, we are aware of various types of meningiomas, and the main types include clival, Convexity, and suprasellar meningiomas (4). The World Health Organization (WHO) has classified meningiomas into three grades: Grade I (81%), Grade II (17%), and Grade III (2%). WHO is low-grade meningiomas, and WHO II and WHO III are high-grade meningiomas, and each grade is recommended to receive its unique treatment (5). The current WHO classification is based on histomorphological features to subclassify meningiomas into 15 subtypes, with nine subtypes assigned to grade I, three to grade II, and the remaining three to grade III (6). Different grades of meningiomas have varying prognoses, with higher grades associated with a higher recurrence rate (I: 7–25%; II: 29–52%; III: 50–94%) and poorer survival outcomes (5). Given these differences in treatment and prognosis, accurate preoperative assessment of tumor grade is clinically necessary to inform treatment decisions.

Early diagnosis of brain tumors improves these patients' chances of survival and helps them recover faster (7). Meningiomas have lower pixel intensity than other brain tumor cells, making them crucial to detect (4). Contrast-enhanced magnetic resonance imaging (MRI) is the initial step in diagnosis; in cases of contraindication, a contrast-enhanced computed tomography (CT) scan is used (8).

We know that the diagnosis of a brain tumor is usually dependent on the manual assessment of the patient by a medical doctor, as well as the test findings of the patient. Particular manual segmentation, often performed in a slice-by-slice method, is too time-consuming to be incorporated into the clinical routine. In this process, doctors may make mistakes because the image patterns of different grades of meningiomas can sometimes mimic each other, resulting in limited diagnostic accuracy and potentially losing valuable treatment time (9). The number of specialist doctors is also restricted; patients may have to wait a long time for a correct diagnosis. However, manual diagnosis of brain tumors from MRI or other usual techniques can be complex, which may lead to inaccurate diagnosis and classification, and this is mainly because brain tumor identification relies on different modules (10). Thus, it is necessary to have improved medical technology in automatic learning to increase the efficiency of specialists, which will reduce the time patients spend in healthcare centers and the amount of time it takes for patients to be diagnosed and recover. Therefore, all these limitations underscore the need for completely automated, deep-learning-based multi-classification systems for brain tumors, including meningioma. Various deep learning-based models, including Convolutional Neural Networks (CNN), NasNet, VGG-16 (developed by the Visual Geometry Group), and Support Vector Machines, have been employed to classify features and yield accurate results (11). They are quickly becoming the primary reference in many fields of health, including medical image analysis (12). Several machine learning (ML) algorithms have been developed for MR image classification, providing radiologists with a new perspective (10).

CNNs are becoming increasingly valuable methods for highly advanced computational histopathology. These tools promote precision medicine through exceptional visual decoding abilities (13). The basic architecture of a CNN consists of a convolutional layer that performs feature extraction and produces feature maps; the next layer is a pooling layer that subsamples these feature maps, and then a fully connected layer that performs classification (12). Rasheed et al. reported that the classification results have demonstrated the ability of deep learning machines to categorize common types of brain tumors with a high level of accuracy (10).

To our knowledge, no research in the literature has conducted a comprehensive review and meta-analysis on the histological grading of meningiomas utilizing deep learning. Consequently, it is essential to perform a systematic review and meta-analysis on this subject, evaluating the quality of the studies and conducting a meta-analysis to quantify the robustness of the existing data. This work aimed to conduct a comprehensive review and meta-analysis of the efficacy of deep learning models in the histological grading of meningioma.

## Method

### Literature search

The PRISMA-DTA (Preferred Reporting Items for Systematic Reviews and Meta-analysis for Diagnostic Test Accuracy) statement was used for this systematic review (14). Before the initiation of the research, the study protocol was registered in the Open Science Framework (OSF) under the DOI number https://doi.org/10.17605/OSF.IO/RXEBM.

Primary publications in the English language that utilize deep learning, machine learning, and/or artificial intelligence in the context of meningioma patients, published between 2000 and 2025, were searched across multiple electronic databases, including Scopus, PubMed, and Web of Science. The search terms consisted of machine learning, artificial intelligence, or deep learning, and meningioma or meningiomas.

The eligible articles underwent full-text evaluation. A third reviewer assisted if a consensus on eligibility was not reached between the two investigators. The search was supplemented by citation analysis and scanning of the reference lists of all eligible articles. Studies were excluded if the article was written in a language other than English, if the majority of patients had brain tumors other than meningiomas, if there was no histologic confirmation of meningioma grading, if sensitivity and specificity were not reported, or if adequate data for their computation were not available (Table 1).

**Table 1:** Curated search strategies and results of the search procedure.

**Database**	**Search strategy**	**Results**
PubMed	(“deep learning”[Title/Abstract] OR “machine learning”[Title/Abstract] OR “Artificial Intelligence”[Title/Abstract] OR “Artificial Intelligence”[MeSH Terms]) AND (“Meningiomas”[Title/Abstract] OR “Meningioma”[Title/Abstract] OR “Meningioma”[MeSH Terms])	289
WOS	((TS=(“deep learning”)) OR TS=(“machine learning”)) OR TS=(“artificial intelligence”)) OR TS=(ai) AND ((TS=(Meningioma)) OR TS=(Meningiomas)	323
Scopus	TITLE-ABS-KEY (“deep learning”) OR TITLE-ABS-KEY (“machine learning”) OR TITLE-ABS-KEY (“artificial intelligence”) OR TITLE-ABS-KEY (ai) AND TITLE-ABS-KEY (meningioma) OR TITLE-ABS-KEY (meningiomas)	194

### Study selection and data extraction

Extracted citations were imported into the Rayyan systematic review platform (https://www.rayyan.ai) for the purpose of study selection. Two independent reviewers screened all titles, abstracts, and full texts for inclusion. Any disagreements were resolved initially through consensus discussions. In cases where consensus could not be reached, a third independent reviewer was used to arbitrate and finalize inclusion decisions. Inter-rater reliability was assessed using Cohen's Kappa statistic (κ), indicating substantial agreement (κ = 0.78).

Data extracted included: (1) first author and year of publication; (2) country (3) mean age and gender of participants in the training set; (4) size of training set; (5) tumor information including volume and location; (6) study design (7) accuracy (8) sensitivity, and specificity for both the training and the internal and/or external test sets (Table 2).

**Table 2:** Summary characteristic of included studies.

**References**	**Year**	**Country**	**Total population**	**Meningioma location**	**DL model**	**DL features (accuracy, sensitivity, specificity, AUC)**	**Findings**
Remzan et al. (7)	2023	Morocco	Not explicitly reported	CPA	CNN classification of brain tumors	Accuracy of 95.65%	Classifier proved to be the most accurate
Prakash et al. (21)	2022	India	82	Left hemisphere	CNN model by tuning the hyper-parameters	Accuracy of 97.39%	It can be applied as a computer-assisted diagnosis
Mahmoud et al. (22)	2023	Egypt	Not explicitly reported	Right hemisphere	VGG-19 model.	Accuracy of 98.95%	Aquila Optimizer (AQO) for accuracy of 98.95% for the VGG-19 model
Priya and Vasudevan (23)	2024	India	Not explicitly reported	Lt and right hemispheres	Proposed hybrid AlexNet-GRU	Accuracy of 97% and Precision of 97.25%	Improve brain tumor detection
Singh et al. (24)	2023	India	149	Lt and right hemispheres	Hybrid Particle Swarm Gray Wolf Optimization (HPSGWO)	99.18% accuracy	Radiologists could use for the first screening for brain tumor classification
Prakash et al. (4)	2023	India	Not explicitly reported	Rt frontal lobe	Proposed HCNN classifier	Accuracy of 99.7% for BRATS 2019 dataset and accuracy of 99.36 for Nanfang dataset	Develop and method for identifying the meningioma
Rasheed et al. (10)	2023	China	233		Proposed CNN	Accuracy (%) of 99.04 and AUC score of 98% and precision, recall, and f1-score success rate of 98%	Can accurately classify various types of brain tumors
Razi et al. (11)	2023	Indonesia	Not explicitly reported	Rt occipital lobe	EfficientNet-B0-B7	EfficientNet-B2 achieves the highest accuracy of 99.9% in training and 99.55% in validation	EfficientNet is a deep learning model that modifies the model so that computational efficiency produces the best results.
Sehring et al. (13)	2023	Germany	193		CNN	Accuracy of 0.870 for benign-1 vs. benign-2 and 0.749 for benign-1	These features can also be made apparent to the human (neuropathologist's) eye
Srinivasan et al. (25)	2024	India	593	Rt hemisphere	Proposed CNN	Accuracy of 99.53%	CNN models were made that can help clinicians; Radiologists check primary screenings
Jun et al. (32)	2023	South Korea	318		DL model that combined the T1C and T2	AUC of 0.770 and accuracy of 72.1% in grading meningiomas	DL model that combined the T1C and T2 can enable fully automatic grading of meningiomas, along with segmentation
Shwetha and Madhavi (33)	2022	India	926	Lt hemisphere	CNN	69% for the first CNN and second model, the accuracy was 71%	Minimize the processing time for tumor images
Mukkapati et al. (34)	2022	India	3,950		CNN with Grid-search hyper parameter	92.98 accuracy	CNN model validates their first screening for multiclassification of brain tumor
Anita et al. (35)	2022	India	1,171	Rt hemisphere	CCNN	98.89% SEIR, 98.74% SPIR, 99.05% AR, 98.93% PR and 98.91% FS	Implement the proposed approaches for the detection of stroke in brain images
Gurunathan et al. (26)	2022	India	1,200	Rt hemisphere	Hybrid CNN- GLCM	accuracy of 99.4%	CNN classifier is proposed and achieves a high classification rate
Chen et al. (36)	2022	China	122	RT hemisphere	ResNet-50	Area under the curve (AUC) of 0.91 and an accuracy of 0.899	Deep learning model to classify preoperative MRI of SFT/HPC and meningioma based on a single T1C
Chen et al. (37)	2021	China	625	RT hemisphere	Deep learning based on radiomics features	Accuracy (training = 0.88//inrernal testing = 086//external testing 0.91)	Deep learning–based segmentation method enables automatic and accurate extraction of meningioma from multiparametric MRimages
Boaro et al. (27)	2022	USA.	936	Longitudinal fissure	3D-CNN	Model achieved a median performance of 88.2%	Deep learning approach to meningioma segmentation is feasible
Anita et al. (35)	2022	India	1,221	Interventricular and rt hemiphere	CNN and segmentation	98.9% of sensitivity (SEN), 99.4% of specificity (SPE), and 99.3% of tumor segmentation accuracy (TSA)	The performance efficiency of the proposed brain tumor detection
Sadoon et al. (38)	2021	Iraq	233	Sphenoid wing and cavernous sinus	CNN	Overall accuracy obtained is 96.1%	Prove the superiority of our model over the rest of the models
Han et al. (5)	2020	China	131	Rt frontal lobe	LR/SVM/RF/DT/KNN AND RADIOMICS	(AUC), 0.956; 95% confidence interval (CI), 0.83–1.00; sensitivity, 0.87; specificity, 0.92; f1-score, 0.90)	The radiomics models are of great value in predicting the histopathological grades of meningiomas
Chen et al. (39)	2021	China	625	Rt hemisphere	3D U-NET SEGMENTATION	area under the curve of 0.918–0.006 and accuracy of 0.901–0.039	Grading models have a promising ability to classify low-grade AND high grade meningiomas
Bouget et al. (40)	2021	Norway	698	Olfactory groove	PLS-Net	F1-score of up to 88%	PLS-Net takes less than a second on GPU and about 15 s on CPU
Hu et al. (41)	2020	china	316	lt hemisphere	Multiparametric radiomic model based on cMRI, ADC map and SWI	Accuracy cMRI + ADC + SWI = 0.78 (0.74–0.83)	Which might offer potential guidance in clinical decision-making
Chen et al. (9)	2019	China	150	Longitudinal fissure	Lasso + LDA	The highest accuracy among LDA-based models was 75.6%, shown in the combination of Lasso + LDA	Could potentially serve as the assistant imaging biomarkers for presurgically grading meningiomas
Banzato et al. (42)	2019	Italy	117	lt hemisphere	V3 DCNN on ADC maps	AUC of 0.94	DCNNs can accurately discriminate between benign and atypical/anaplastic meningiomas from ADC maps but not from PCT1W images
Hale et al. (43)	2018	USA	128		Optimized ANN; Optimized k-NN	ANN (AUC = 0.8895); k-NN models (AUC = 0.8687)	ML algorithms are powerful computational tools that can predict meningioma grade
Remzan et al. (7)	2023	Morocco	Not explicitly reported	CPA	CNN classification of brain tumors	Accuracy of 95.65%.	Classifier proved to be the most accurate
Prakash et al. (21)	2022	India	82	Left hemisphere	CNN model by tuning the hyper-parameters	Accuracy of 97.39%	It can be applied as a computer-assisted diagnosis.
Mahmoud et al. (22)	2023	Egypt	Not explicitly reported	Right hemisphere	VGG-19 model.	accuracy of 98.95%	Aquila Optimizer (AQO) for accuracy of 98.95% for the VGG-19 model
Priya and Vasudevan (23)	2024	India	Not explicitly reported	Lt and right hemispheres	Proposed hybrid AlexNet-GRU	accuracy of 97% and Precision of 97.25%	Improve brain tumor detection.
Singh et al. (24)	2023	India	149	Lt and right hemispheres	Hybrid Particle Swarm Gray Wolf Optimization (HPSGWO)	99.18% accuracy	radiologists could use for the first screening for brain tumor classification.
Prakash et al. (4)	2023	India	Not explicitly reported	Rt frontal lobe	Proposed HCNN classifier	accuracy of 99.7% for BRATS 2019 dataset and accuracy of 99.36 for Nanfang dataset	Develop and method for identifying the meningioma
Rasheed et al. (10)	2023	China	233		Proposed CNN	ACCURACY(%) of 99.04 and AUC score of 98% and precision, recall, and f1-score success rate of 98%,	Can accurately classify various types of brain tumors
Razi et al. (11)	2023	Indonesia	Not explicitly reported	Rt occipital lobe	EfficientNet-B0-B7	EfficientNet-B2 achieves the highest accuracy of 99.9% in training and 99.55% in validation.	EfficientNet is a deep learning model that modifies the model so that computational efficiency produces the best results.
Sehring et al. (13)	2023	Germany	193		CNN	Accuracy of 0.870 for benign-1 vs. benign-2 and 0.749 for benign-1	These features can also be made apparent to the human (neuropathologist's) eye
Srinivasan et al. (25)	2024	India	593	Rt hemisphere	Proposed CNN	Accuracy of 99.53%	CNN models were made that can help clinicians; Radiologists check primary screenings
Jun et al. (32)	2023	South Korea	318		DL model that combined the T1C and T2	AUC of 0.770 and accuracy of 72.1% in grading meningiomas	DL model that combined the T1C and T2 can enable fully automatic grading of meningiomas, along with segmentation
Shwetha and Madhavi (33)	2022	India	926	Lt hemisphere	CNN	69% for the first CNN and second model, the accuracy was 71%	Minimize the processing time for tumor images
Mukkapati et al. (34)	2022	India	3,950		CNN with Grid-search hyper parameter	92.98 accuracy	CNN model validates their first screening for multiclassification of brain tumor
Anita et al. (35)	2022	India	1,171	Rt hemisphere	CCNN	98.89% SEIR, 98.74% SPIR, 99.05% AR, 98.93% PR and 98.91% FS	Implement the proposed approaches for the detection of stroke in brain images
Gurunathan et al. (26)	2022	India	1,200	Rt hemisphere	Hybrid CNN- GLCM	Accuracy of 99.4%	CNN classifier is proposed and achieves a high classification rate
Chen et al. (36)	2022	China	122	RT hemisphere	ResNet-50	Area under the curve (AUC) of 0.91 and an accuracy of 0.899	Deep learning model to classify preoperative MRI of SFT/HPC and meningioma based on a single T1C
Chen et al. (37)	2021	China	625	RT hemisphere	Deep learning based on radiomics features	Accuracy (training = 0.88//inrernal testing = 086//external testing 0.91)	Deep learning–based segmentation method enables automatic and accurate extraction of meningioma from multiparametric MRimages
Boaro et al. (27)	2022	USA.	936	Longitudinal fissure	3D-CNN	model achieved a median performance of 88.2%	Deep learning approach to meningioma segmentation is feasible
Anita et al. (35)	2022	India	1,221	Interventricular and rt hemiphere	CNN and segmentation	98.9% of sensitivity (SEN), 99.4% of specificity (SPE), and 99.3% of tumor segmentation accuracy (TSA)	The performance efficiency of the proposed brain tumor detection
Sadoon et al. (38)	2021	Iraq	233	Sphenoid wing and cavernous sinus	CNN	Overall accuracy obtained is 96.1%.	Prove the superiority of our model over the rest of the models
Han et al. (5)	2020	China	131	Rt frontal lobe	LR/SVM/RF/DT/KNN AND RADIOMICS	(AUC), 0.956; 95% confidence interval (CI), 0.83–1.00; sensitivity, 0.87; specificity, 0.92; f1-score, 0.90)	The radiomics models are of great value in predicting the histopathological grades of meningiomas
Chen et al. (39)	2021	China	625	Rt hemisphere	3D U-NET SEGMENTATION	Area under the curve of 0.918–0.006 and accuracy of 0.901–0.039	Grading models have a promising ability to classify low-grade AND high grade meningiomas
Bouget et al. (40)	2021	Norway	698	Olfactory groove	PLS-Net	F1-score of up to 88%	PLS-Net takes less than a second on GPU and about 15 s on CPU
Hu et al. (41)	2020	China	316	lt hemisphere	Multiparametric radiomic model based on cMRI, ADC map and SWI	Accuracy cMRI + ADC + SWI =0.78 (0.74–0.83)	Which might offer potential guidance in clinical decision-making
Chen et al. (9)	2019	China	150	Longitudinal fissure	Lasso + LDA	The highest accuracy among LDA-based models was 75.6%, shown in the combination of Lasso + LDA	Could potentially serve as the assistant imaging biomarkers for presurgically grading meningiomas
Banzato et al. (42)	2019	Italy	117	lt hemisphere	V3 DCNN on ADC maps	AUC of 0.94	DCNNs can accurately discriminate between benign and atypical/anaplastic meningiomas from ADC maps but not from PCT1W images
Hale et al. (43)	2018	USA	128		optimized ANN; Optimized k-NN	ANN (AUC = 0.8895); k-NN models (AUC = 0.8687)	ML algorithms are powerful computational tools that can predict meningioma grade

### Quality assessment

For each selected publication, we extracted the following information: first author, year of publication, study population (number of patients, sex, age, and histology), meningioma grade and location, DL model, DL features (accuracy, sensitivity, specificity, and AUC), and findings. When possible, data were recorded at the patient level. The quality of primary studies was assessed using the Ottawa-Newcastle Scale for quality assessment by two independent reviewers. Any disagreements between the two reviewers were resolved by mutual consensus and then independently scored by a third reviewer. The meta-analysis included all studies with low concerns regarding applicability in the three domains (patient selection, index test, and reference standard) (Table 3).

**Table 3:** Results of New Castle Ottawa quality assessment of studies.

**References**	**Selection**				**Comparability of cases and controls on the basis of the design or analysis**	**Exposure**			**Final score**
	**In this case definition adequate**	**Representativeness of the cases**	**Selection of controls**	**Definition of controls**		**Ascertainment of exposure**	**Same method of ascertainment for cases and controls**	**None response rate**	
Srinivasan et al. (25)	^*^		^*^	^*^		^*^	^*^	^*^	6
Remzan et al. (7)	^*^	^*^	^*^	^*^		^*^	^*^	^*^	7
Priya and Vasudevan et al. (23)	^*^	^*^	^*^	^*^		^*^	^*^	^*^	7
Mahmoud et al. (22)	^*^	^*^	^*^	^*^		^*^	^*^	^*^	7
Singh et al. (24)	^*^		^*^	^*^		^*^	^*^	^*^	6
Prakash et al. (4)	^*^	^*^	^*^	^*^		^*^	^*^		6
Rasheed et al. (10)	^*^		^*^	^*^		^*^	^*^	^*^	6
Razi et al. (11)	^*^		^*^	^*^		^*^	^*^	^*^	6
Sehring et al. (13)	^*^	^*^	^*^	^*^	^*^	^*^	^*^	^*^	8
Prakash et al. (21)	^*^		^*^	^*^		^*^	^*^	^*^	6
Jun et al. (32)	^*^	^*^	^*^	^**^	^*^	^*^	^*^	^*^	9
Shwetha and Madhavi (33)	^*^		^*^	^*^	^*^	^*^	^*^	^*^	7
Mukkapati et al. (34)	^*^		^*^	^*^		^*^	^*^	^*^	6
Anita et al. (35)	^*^	^*^	^*^	^*^		^*^	^*^	^*^	7
Gurunathan et al. (26)	^*^	^*^	^*^		^*^	^*^	^*^	^*^	7
Chen et al. (36)	^*^	^*^	^*^	^**^	^*^	^*^	^*^		8
Chen et al. (37)	^*^	^*^	^*^	^**^	^*^	^*^	^*^		8
Boaro et al. (27)	^*^		^*^	^*^	^*^	^*^	^*^		6
Anita et al. (35)	^*^	^*^	^*^	^*^	^*^	^*^	^*^	^*^	8
Sadoon et al. (38)	^*^		^*^	^*^	^*^	^*^	^*^	^*^	6
Han et al. (5)	^*^		^*^		^**^	^*^	^*^	^*^	7
Chen et al. (39)	^*^		^*^	^*^	^*^	^*^	^*^		6
Bouget et al. (40)	^*^	^*^	^*^	^*^	^**^	^*^	^*^	^*^	9
Hu et al. (41)	^*^	^*^	^*^	^*^	^**^	^*^	^*^	^*^	9
Chen et al. (9)	^*^	^*^	^*^	^**^	^*^	^*^			7
Banzato et al. (42)	^*^		^*^	^*^	^*^	^*^	^*^	^*^	7
Hale et al. (43)	^*^	^*^	^*^	^*^	^**^	^*^	^*^	^*^	9

Each astrikes refer to one score the study received after quality assessment.

### Statistical analyses

A meta-analysis was conducted to calculate a pooled estimate of the sensitivity, specificity, and area under the curve (AUC) as measures of the performance of deep learning models in histopathological grading of meningiomas. Heterogeneity was assessed using the Chi-square and I-square tests.

Statistical heterogeneity was assessed using the I^2^ value, which indicates the percentage of variability across the included studies. The rate of difference between studies that can be attributed to heterogeneity as opposed to chance is expressed by the I^2^ statistic. I^2^ was computed as follows: 100% × (Q-df)/Q equals I^2^. The inverse variance approach was used to determine the weight of each study. A random-effects model was used to estimate the overall impact size by combining data from all included studies. This method helped reduce two-sided study heterogeneity values, and *p* ≤ 0.05 were deemed statistically significant.

A subgroup analysis was performed to investigate the factors contributing to heterogeneity. Data points from graphical representations in studies were extracted using WebPlot Digitizer (Automeris LLC, Frisco, Texas).

### Publication bias assessment

The study examined publication bias using Egger's regression. When Egger's regression identified significant bias (*P* < 0.05), a trim and fill analysis was performed to estimate the potential missing effect sizes and determine a revised overall effect (15).

### Sensitivity analysis

Additionally, a sensitivity analysis was conducted on the meta-analysis results using the one-study-removed method to assess the impact of a specific study on the overall estimation of effects (16).

### Bivariate SROC analysis

To summarize the diagnostic performance of the included AI models, we performed a bivariate random-effects meta-analysis of sensitivity and specificity. This approach accounts for both within-study variability and the inherent correlation between sensitivity and specificity across studies. From each eligible study, we extracted point estimates of sensitivity and specificity and plotted them in the receiver operating characteristic (ROC) space. The bivariate model was then used to fit a summary receiver operating characteristic (SROC) curve and to estimate the summary sensitivity, specificity, and 95% confidence interval. This model was selected by current recommendations for meta-analyses of diagnostic test accuracy studies, as it provides a more reliable synthesis of diagnostic performance compared to univariate or simple pooling methods. The resulting SROC curve visually illustrates the trade-off between sensitivity and specificity across studies, as well as the overall discriminative ability of the AI models.

## Results

### Study selection

**Figure 1:**
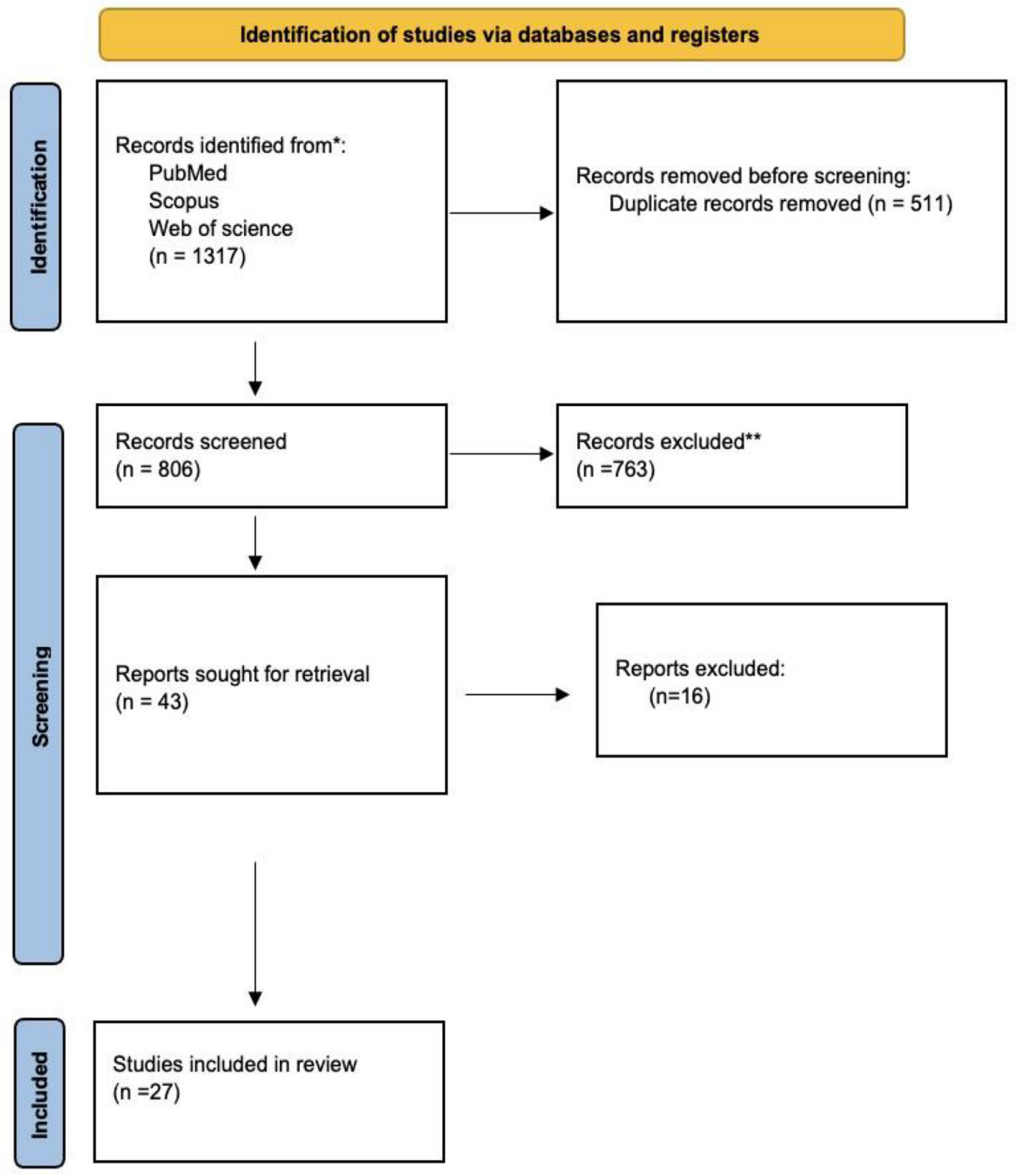
PRISMA-DTA flowchart of the study selection procedure.

We identified 1,317 studies in PubMed, Web of Science, and Scopus based on the keyword combination search. After eliminating 511 duplicates, 806 articles underwent screening based on their titles and abstracts. Seven hundred sixty-three studies were excluded due to the inappropriateness of the study design, irrelevance, or inaccessibility to the full text. Twenty-seven publications were ultimately selected for inclusion in our analysis (Figure 1).

This investigation involved a total of 13,130 participants. According to the data analysis, 5,454 and 3,109 participants were included in the training set and test set groups, respectively. The mean age for the total participants was 52.3 ± 4.2 years. The mean ages of the test set and training set were 50.47 ± 4.2 and 50.94 ± 3.2 years, respectively. Fourteen studies were conducted in India, 13 in China, 5 in Morocco, 4 in Egypt, 3 in Korea, and one in Singapore (Table 3).

### Meta-analysis

**Figure 2:**
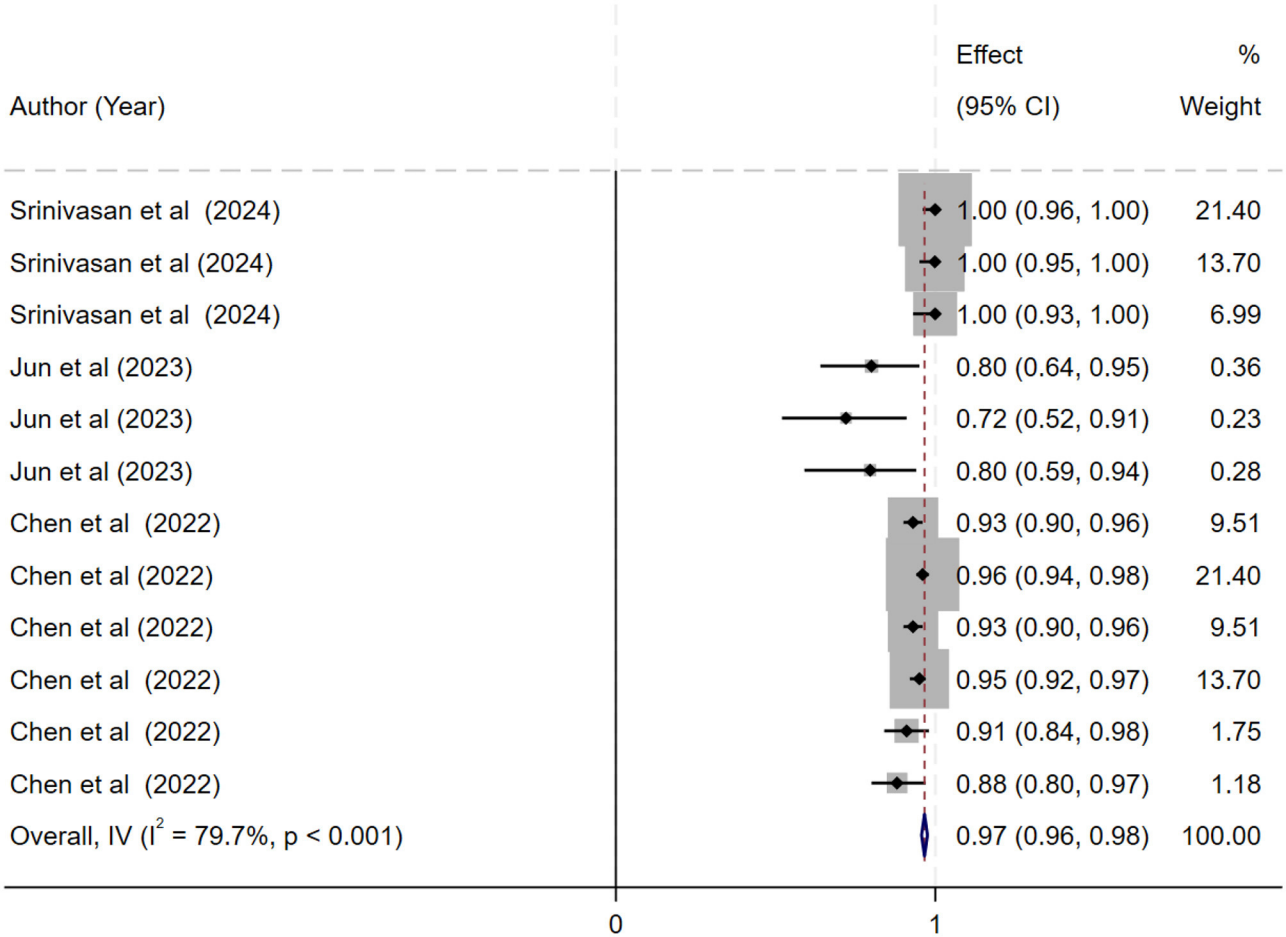
Forest plot showing the pooled area under the curve (AUC) for deep learning models in the histopathological grading of meningiomas. Each horizontal line represents an individual study's AUC with 95% confidence intervals, labeled with the first author and publication year. The black diamond indicates the overall pooled AUC (0.97; 95% CI, 0.96–0.98) calculated using a random-effects model. Data labels include sample sizes and key performance metrics where available.

**Figure 3:**
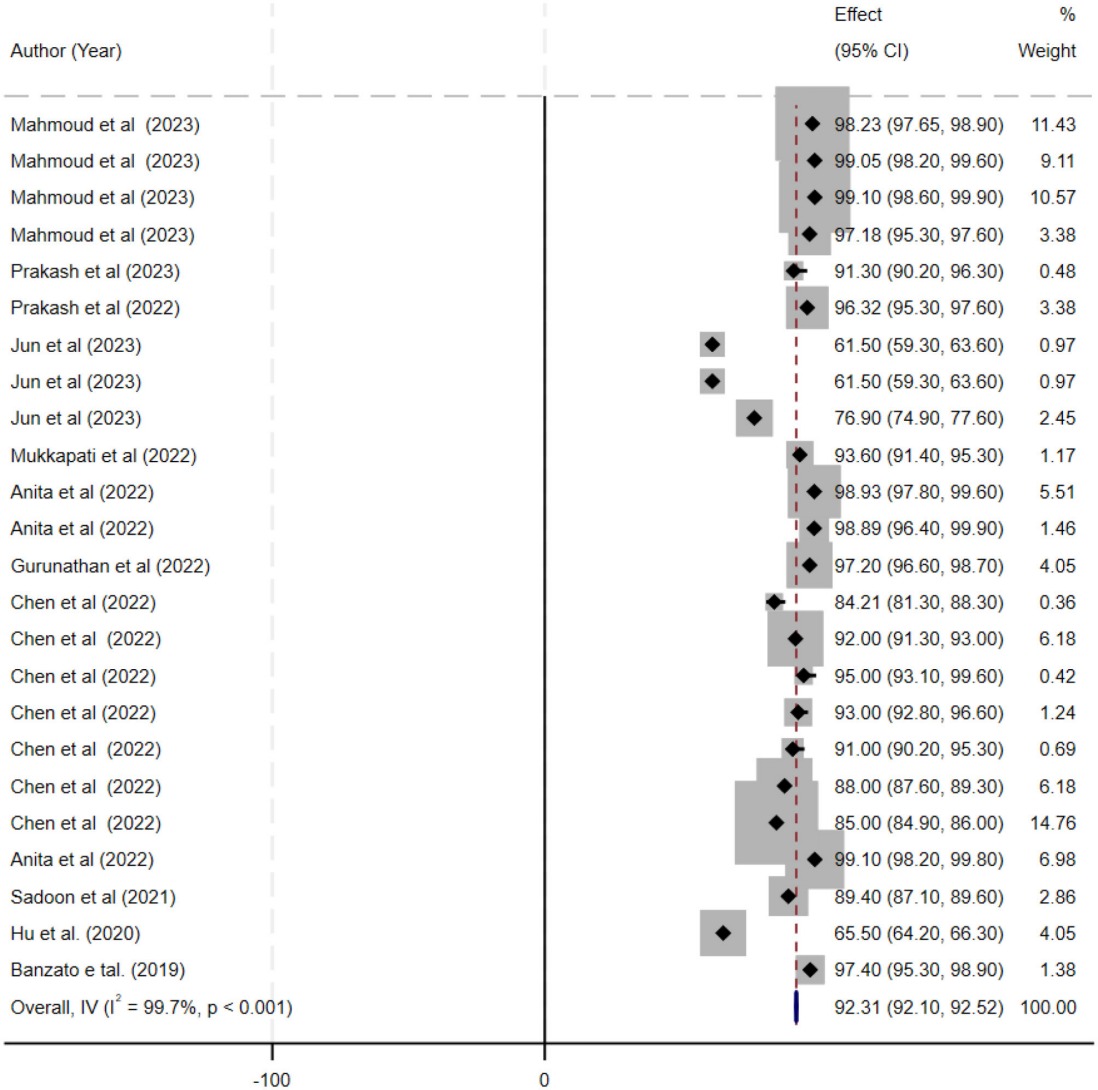
Forest plot displaying the sensitivity estimates of individual deep learning models for meningioma grading. Each study's sensitivity (with corresponding 95% confidence intervals) is represented by a horizontal line and is annotated with study details (first author, year, and sample size). The overall pooled sensitivity is shown as a black diamond on the x-axis (ranging from 0 to 1).

**Figure 4:**
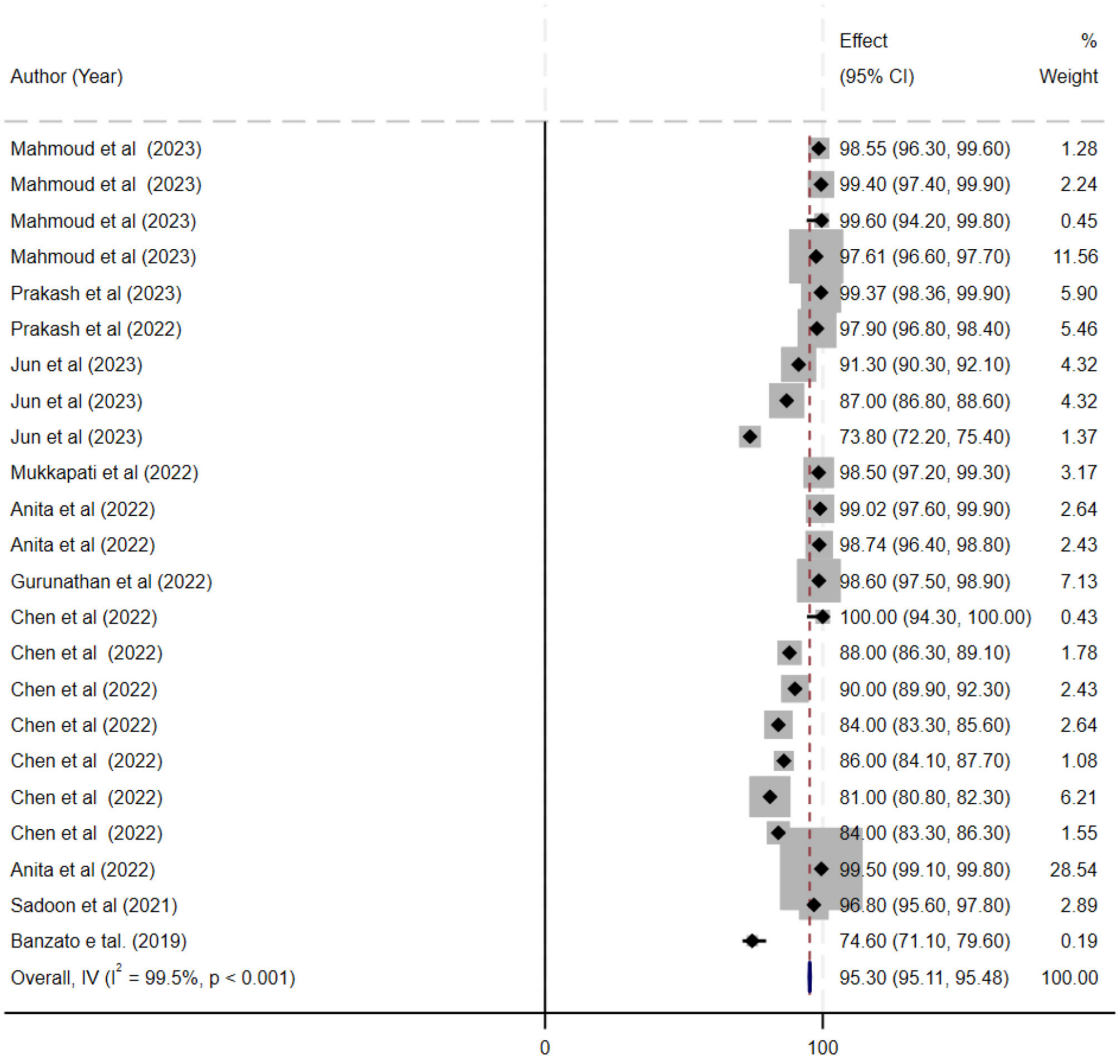
This figure presents a forest plot of specificity estimates for deep learning models in the grading of meningiomas. Individual study specificity values are shown with 95% confidence intervals, and each study is identified by the first author and year, with sample size details provided. The overall pooled specificity is indicated by a black diamond on the x-axis.

**Figure 5:**
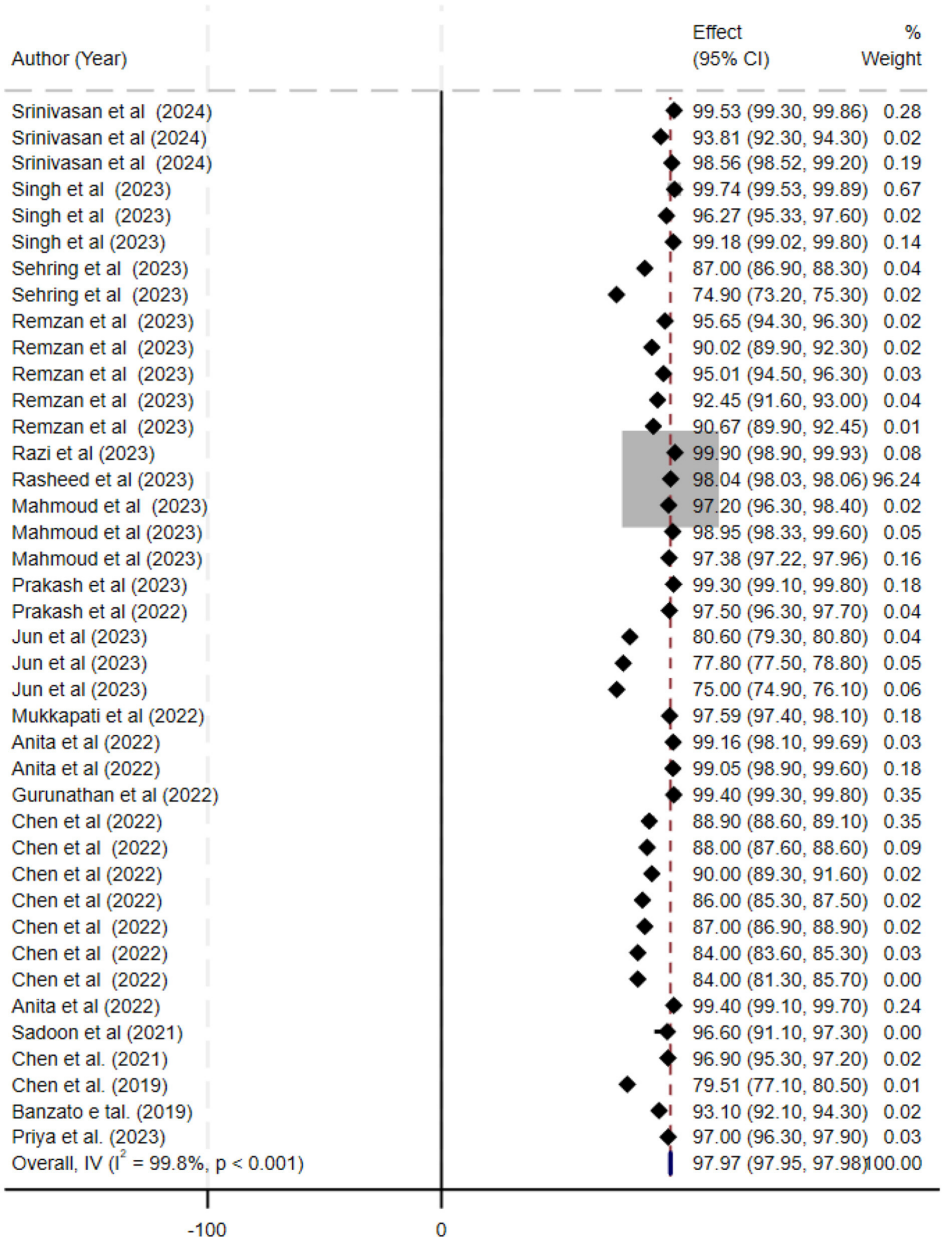
Forest plot summarizing the accuracy of deep learning models for the automatic histopathological grading of meningiomas. Each line represents a study's accuracy estimate with 95% confidence intervals, annotated with the corresponding study (first author, year) and sample size. The black diamond denotes the overall pooled accuracy (97.97%; 95% CI, 97.35–97.98). The x-axis displays accuracy percentages, and a footnote describes that a random-effects model was used for pooling the data and that heterogeneity was assessed statistically.

**Figure 6:**
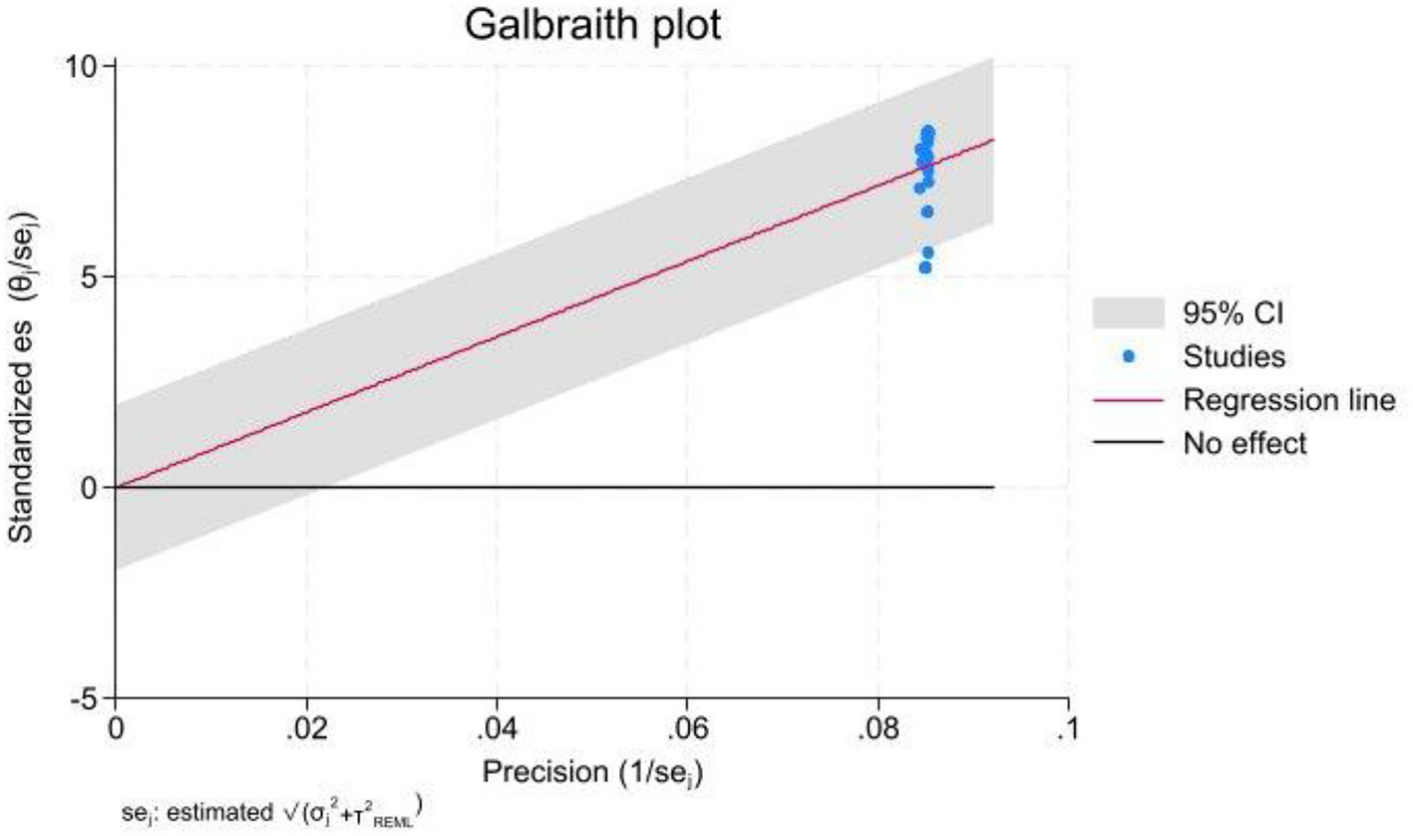
Galbraith plot used to evaluate heterogeneity among the included studies. Standardized effect sizes are plotted against their precision, with each point representing a study, labeled with the first author and publication year. Dashed lines indicate the expected 95% limits of variation. A legend explains the symbols, and an annotation at the bottom notes that studies falling outside these lines contribute to the significant heterogeneity observed.

The ML models for meningioma characterization yielded an overall pooled AUC of 0.97 (95% CI, 0.96–0.98) (Figure 2). Study heterogeneity was 79.7% (*p* < 0.001). The pooled accuracy, sensitivity, and specificity of the models were calculated as follows: sensitivity, 92.31 (95% CI: 92.1–92.52); specificity, 95.3 (95% CI: 95.11–95.48); and accuracy, 97.97 (95% CI: 97.35–97.98) (Figures 3–6).

### Diagnostic test accuracy analysis

**Figure 7:**
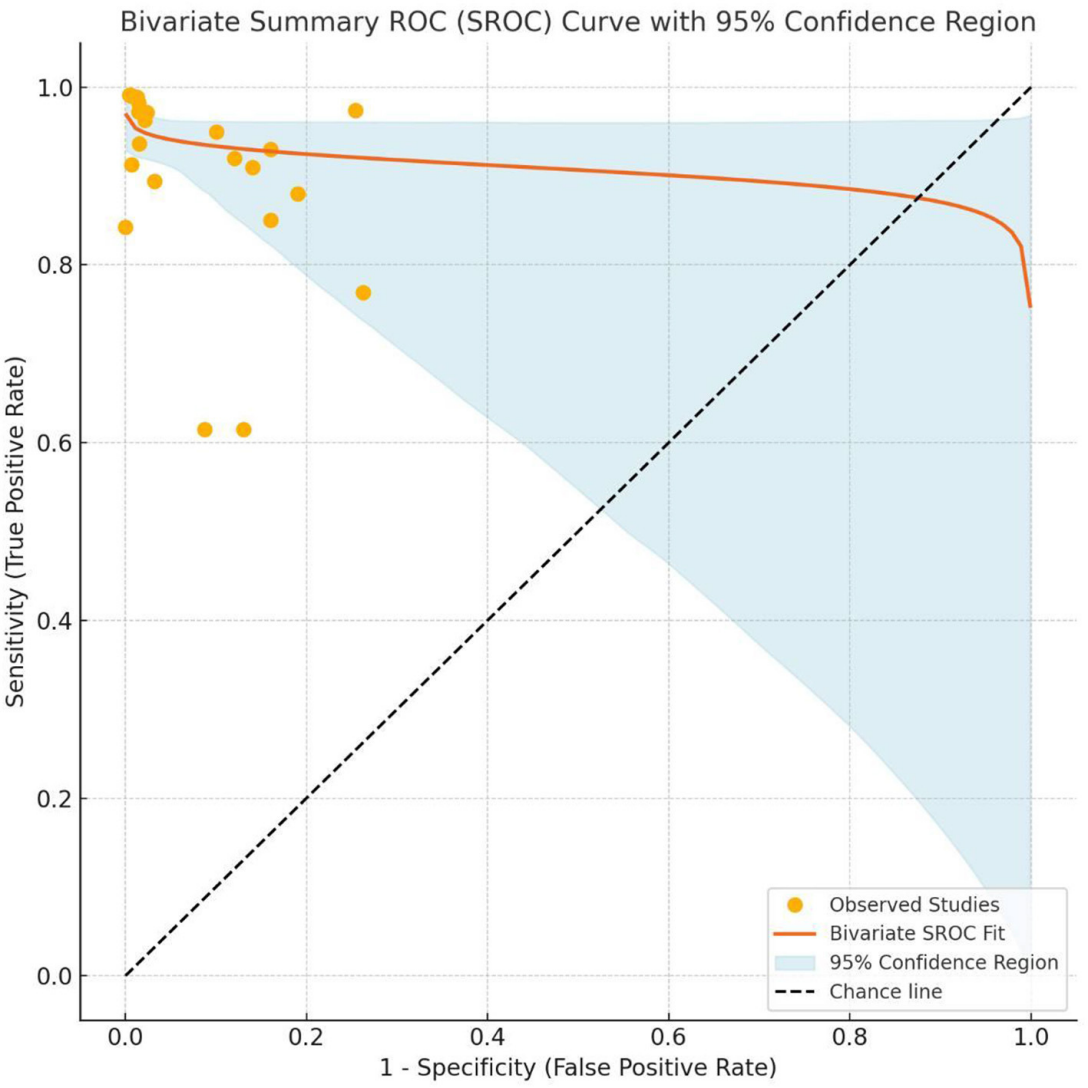
Bivariate Summary Receiver Operating Characteristic (SROC) Curve with 95% confidence region for AI-based diagnostic performance. Each circle represents an individual study reporting sensitivity and specificity of AI models in the detection task. The solid curve illustrates the bivariate SROC curve summarizing the diagnostic performance across studies. The light blue shaded area represents the 95% confidence region, indicating the uncertainty around the pooled estimate. The diagonal dashed line indicates the chance line (AUC = 0.5). The curve's proximity to the upper left corner reflects high diagnostic accuracy of the AI models evaluated in the meta-analysis.

The bivariate summary receiver operating characteristic (SROC) curve (Figure 7) provides a comprehensive overview of the diagnostic accuracy of the included AI models across studies. The curve demonstrates a favorable balance between sensitivity and specificity, as most studies are concentrated in the upper-left quadrant of the ROC space, reflecting consistently high diagnostic performance. The fitted bivariate SROC curve displayed a smooth trajectory, indicating a stable relationship between sensitivity and specificity, despite variations in study populations, AI architectures, and imaging modalities. The accompanying 95% confidence region was relatively narrow, indicating moderate heterogeneity among studies but supporting the reliability of the pooled diagnostic performance. Collectively, these findings suggest that AI-based models exhibit robust diagnostic accuracy when applied to [insert your diagnostic task] and may be considered promising tools for assisting clinical decision-making.

### Publication bias

**Figure 8:**
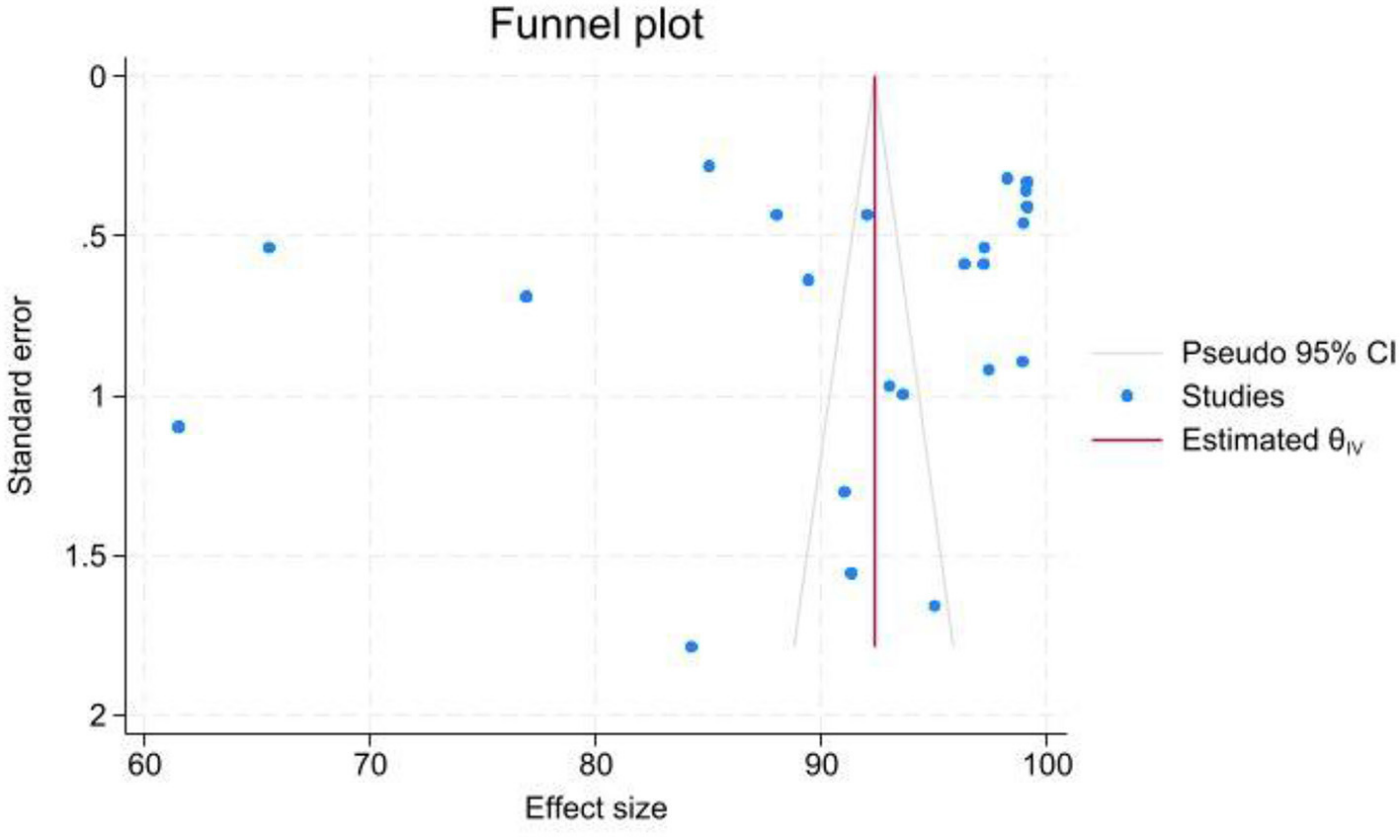
Funnel plot assessing the presence of publication bias among the studies included in the meta-analysis. The x-axis represents the effect sizes (AUC values) and the y-axis shows the standard errors. Each data point is annotated with study identifiers (first author, year) and sample sizes. The asymmetry observed in the plot suggests potential publication bias, which is further supported by Egger's test (*p*-value slightly > 0.05). A legend clarifies the symbols used, and a reference line indicates the overall pooled effect size.

The funnel plot and Egger's test were performed to investigate the possible publication bias. The funnel plot exhibited an asymmetrical pattern, suggesting the presence of publication bias at some levels; this was further confirmed by Egger's test, which yielded a *p*-value slightly higher than 0.05 (Figure 8).

## Discussion

### Overview of study findings

This systematic review and meta-analysis evaluated the performance of various deep learning (DL) models for the automatic histopathological grading of meningiomas. The reviews in this study demonstrate the remarkable improvements and high classification accuracy rates achieved by several deep learning (DL) models in meningioma grading. Considering the complexities of histopathological images, these models indicate significant promise for assisting pathologists and enhancing the accuracy of diagnosis.

### Histopathological grading and AI

Compared to radiologic studies, there have been fewer recent publications applying deep learning directly to histopathological slides for meningioma grading. Since grading criteria (mitotic count, brain invasion, etc.) are assessed on microscope slides by pathologists, the role of AI here is often to assist rather than replace the expert.

AI tools are being developed to support pathologists—for example, automated mitosis detection systems (initially developed for other tumors) could be adapted to quantify mitotic figures in meningiomas, aiding grade determination. Some integrated approaches also use imaging to anticipate histologic features: as noted, modern DL models can predict markers like Ki-67 index from MRI? (17), which correlates with tumor grade and aggressiveness. In essence, most “AI grading” research for meningiomas focuses on pre-surgical radiological prediction; once tissue is obtained, standard histopathologic grading, possibly with AI-assisted quantification, is the gold standard. We anticipate that future studies will apply deep learning to whole-slide images to classify meningioma subtypes or distinguish between grade II and III features.

### Comparative analysis of models

Recent studies collectively shed light on the strengths and weaknesses of different AI approaches for meningioma grading:

CNN vs. traditional ML: deep CNN-based models automatically learn complex image patterns and tend to achieve higher sensitivity, capturing subtle signs of malignancy, whereas traditional feature-based models can be more specific, resulting in fewer false alarms. Performance metrics (AUC ~0.8–0.93) overlap considerably (18), so the choice may depend on data availability and the need for interpretability. Notably, adding hand-crafted radiomics to deep models (a hybrid approach) can further boost accuracy in some cases (19). Architectures and Innovations: Vision Transformers (ViT), ensemble CNNs, and advanced training techniques, including transfer learning, data augmentation, and hyperparameter tuning, have been explored to enhance grading performance. For example, incorporating ViT to capture global image context alongside CNN-localized features led to an accuracy of over 92% in one study (18). Ensembling or comparing multiple architectures (ResNet, DenseNet, VGG, etc.) has reaffirmed that carefully optimized CNNs outperform older networks for brain tumor classification (19). However, beyond a certain accuracy level, gains become incremental; attention shifts toward ensuring models are robust and generalize well, rather than simply increasing in-sample accuracy.

Internal vs. external validation: a consistent finding is that models must be tested on external data. Smaller single-center studies often report impressively high AUC/accuracy internally; however, as seen, these results may not fully hold up in new patient cohorts. Multi-institutional datasets and prospective validations are crucial. The NPJ Precision Oncology study, a multi-hospital study, is a positive example, demonstrating nearly equal performance on both internal and external sets (AUC ~0.80). In contrast, models without such diverse training can suffer sizable drops in external metrics (e.g., 97% → 67% AUC). Therefore, when comparing model reports, clinicians should weigh whether the evaluation included an independent test cohort—a model with slightly lower reported accuracy but proven on external data may be more clinically reliable than one with sky-high accuracy on a narrow sample (20).

Sensitivity, specificity, and AUC: most deep learning models for meningioma grading achieve sensitivity and specificity in the 75–90% range on test sets. For instance, one automatic DL segmentation + radiomics model yielded ~78% sensitivity and 86% specificity (AUC ~0.84) on its test cohort, while a pure DL classifier might reach >90% on internal data but lower on external. The clinical significance of these numbers is important: a sensitivity in the upper 80s means the majority of higher-grade tumors would be correctly flagged before surgery, potentially prompting more aggressive resection or closer follow-up. Specificity in a similar range means relatively few benign tumors would be misclassified as high-grade (avoiding undue alarm or overtreatment). An AUROC around 0.8–0.9 indicates good discriminative ability, though not perfect. Generally, these metrics are high enough to be clinically useful as a decision-support tool, but not to replace definitive histopathology (20).

### Accuracy and performance

The deep learning models demonstrated impressive accuracy rates across the studies. For instance, a CNN model achieved an accuracy of 95.65% in classifying brain tumors, indicating its robustness in handling complex histopathological images (7). Another study reported an even higher accuracy of 97.39% using a CNN model with optimized hyperparameters, showcasing the benefits of fine-tuning these models to enhance performance (21). A notable performance was seen with a VGG-19 model, which achieved an accuracy of 98.95% using the Aquila Optimizer (AQO) (22). This result emphasizes the potential of advanced optimization techniques in improving model performance. Furthermore, a proposed hybrid AlexNet-GRU model showed an accuracy of 97% and a precision of 97.25%, suggesting its potential utility in clinical settings (23). The highest accuracy for distinguishing different brain tumor grades reported was 99.18%, achieved by a Hybrid Particle Swarm Gray Wolf Optimization (HPS-GWO) model, underscoring the model's exceptional performance (24).

### Model-specific performance

#### Convolutional neural network (CNN) and combination with CNN models

The CNN model evaluated in Remzan et al.'s study demonstrated a high accuracy of 95.65% in classifying meningiomas, showcasing its effectiveness in recognizing complex patterns within histopathological images. This high level of accuracy indicates that CNNs, when adequately trained, can be reliable tools for grading meningiomas. The robustness of CNNs is further supported by their ability to handle large amounts of data and automatically extract relevant features (7).

Prakash et al. reported a CNN model with optimized hyperparameters that achieved an accuracy of 97.39%. The optimization of hyperparameters likely contributed to the enhanced performance, indicating that fine-tuning deep learning (DL) models can significantly improve their accuracy and reliability in clinical applications. This finding suggests that continuous improvements and adjustments in model parameters are crucial for achieving optimal results in medical image analysis (21).

To distinguish between meningioma and non-meningioma brain images, a novel hybrid convolutional neural network (HCNN) classifier is presented by B. V. Prakash. Several components are integrated into the HCNN classification technique, including a segmentation algorithm, a classifier module, a Ridgelet transform, and feature computation. The features are obtained from the Ridgelet coefficients, and the Ridgelet transform improves pixel stability during the decomposition process. This novel strategy aims to enhance the accuracy and reliability of brain image classification in medical diagnosis (4).

Rasheed et al. demonstrate the reliability of the proposed system by achieving exact prediction results, which compare favorably to those of previous studies of a similar nature. The proposed CNN method is a segment-free approach that directly loads images of brain tumors to obtain tumor classifications. In comparison, alternative approaches require additional manual steps, such as tumor localization or feature extraction (10).

Sehring et al. introduce an AI-assisted image analysis approach that utilizes the visual decoding capabilities of convolutional neural networks (CNNs) to identify prognostically relevant, methylome-defined tumor classes of meningiomas using conventional HE-stained histopathology slides. The primary objective was to predict methylation classes in meningiomas based on histological features through a deep-learning framework. This task surpasses the capabilities of neuropathologists, as DNA methylation-based molecular classification has shown higher predictive power for tumor recurrence compared to histopathological classification alone (13).

Using CNN models, Srinivasan et al. propose a multi-classification approach for early-stage brain tumor diagnosis, where nearly all hyperparameters are automatically adjusted through a grid search. Using publicly available medical imaging datasets, three reliable CNN models were designated for different brain tumor classification tasks. These models achieved high levels of accuracy in detecting brain tumors, classifying brain MR images into various categories, and grading glioma brain tumors (25).

A CNN-based deep net approach to the detection and classification of meningioma brain tumors is proposed by Gurunathan et al. To extract deep features from input brain MRI images, the CNN Deep Net architecture comprises five convolutional layers with ReLU activations, Max pooling layers, and a multi-neuron feedforward neural network. A multilayer perceptron architecture is utilized to detect and classify brain cancers. The detected tumor-affected regions are segmented using a global threshold segmentation approach, with tumor sites located using dilation and erosion techniques. Furthermore, a new diagnostic system with a high classification rate and accuracy is suggested, utilizing a GLCM CNN classifier (26).

Boaro et al. introduce a three-dimensional convolutional neural network (3D-CNN) capable of performing expert-level automated segmentation and volumetric assessment of meningiomas in MRI scans. The 3D-CNN was initially trained to segment whole-brain volumes using a dataset of 10,099 healthy brain MRIs. The resultant model achieved a median performance of 88.2%, which fell within the existing inter-expert variability range of 82.6–91.6%. The work demonstrates that a deep learning strategy for meningioma segmentation is viable, extremely precise, and may improve existing therapeutic practices in a simulated clinical setting (27).

#### VGG-19 model

Mahmoud et al.'s study highlighted the performance of a VGG-19 model, which achieved an accuracy of 98.95% with the assistance of the Aquila Optimizer (AQO). This result suggests that the VGG-19 model, when combined with advanced optimization techniques, can substantially enhance classification performance, making it a strong candidate for clinical use. The VGG-19 model's deep architecture, which includes multiple layers, enables the extraction of detailed features from images, thereby enhancing its diagnostic capabilities (22).

#### Hybrid AlexNet-GRU model

The hybrid AlexNet-GRU model proposed by Priya et al. showed an accuracy of 97% and a precision of 97.25%. This model's performance underscores the potential benefits of combining different neural network architectures to capitalize on their strengths, leading to improved accuracy and precision in grading meningiomas. Integrating AlexNet's convolutional layers with GRU's recurrent layers enables the model to capture spatial and temporal features, which is particularly beneficial for analyzing complex histopathological images (23).

#### Hybrid Particle Swarm Gray Wolf Optimization (HPS-GWO) model

Singh et al. evaluated the HPS-GWO model, which achieved the highest accuracy of 99.18%. This model's exceptional performance highlights the potential of hybrid optimization algorithms in enhancing the accuracy of deep learning models. The use of particle swarm optimization and gray wolf optimization techniques likely contributed to the model's superior performance, making it a promising tool for clinical implementation. Combining these optimization techniques enables efficient exploration of the solution space and convergence to optimal solutions, thereby enhancing the model's accuracy (24).

### Systematic comparison of deep learning models for meningioma grading

Based on our systematic review, we identified five main categories of deep learning (DL) models applied in meningioma grading (Table 4).

**Table 4:** Comparative summary of deep learning model categories for meningioma grading.

**Model**	**Strengths**	**Limitations**	**Suitable scenarios**
Convolutional neural networks (CNN)	High accuracy (>95% in most studies); automatic feature extraction; adaptable to various imaging types	Sensitive to dataset quality and imbalance; sometimes lacks interpretability.	Standard meningioma classification from MRI and histopathological images
VGG Variants (e.g., VGG-16, VGG-19)	Deeper networks improve feature extraction; strong performance when combined with optimizers (Aquila Optimizer)	Computationally expensive; prone to overfitting if the dataset is small	Complex feature extraction and cases where rich image information is available
Hybrid Models (e.g., AlexNet-GRU, CNN-GLCM)	Combines spatial and sequential feature extraction to improve accuracy and robustness.	Increased model complexity; requires more computational resources	Cases involving temporal or sequential data or complex tumor morphology
3D-CNN Models	Effective for volumetric and 3D imaging; good for segmentation and grading	Needs large annotated datasets; high computational cost	Tumor segmentation and volume-based grading in preoperative imaging
Optimization-enhanced Models (e.g., HPS-GWO, Ridgelet-CNN)	Achieves highest reported accuracies (>99%); robust against image variations	Limited generalizability without external validation; requires hyperparameter tuning	Specialized centers with access to optimized imaging pipelines and computational power

This comparative analysis highlights that while CNN-based models are reliable and widely used, hybrid and optimization-enhanced models exhibit superior performance, albeit at the cost of increased complexity and resource demand. The choice of model should therefore balance between available computational resources, clinical needs (classification vs. segmentation), and dataset size.

The reported high AUC of 0.97 in many studies raises concerns that these models might be overfitting the training data. Overfitting occurs when a model learns the noise and specific patterns in a limited dataset rather than the underlying generalizable features. In this context, an unusually high AUC may not reflect true predictive power when applied to new, unseen data. This phenomenon is particularly concerning when the datasets are small or when there is little external validation. Without rigorous cross-validation and testing on diverse patient cohorts, the performance metrics might be overly optimistic, risking poor generalizability in real-world clinical settings.

In addition, funnel plot asymmetry observed in some analyses suggests the possibility of publication bias, where studies with negative or less impressive results are underreported or unpublished. This can lead to an inflated overall effect size, as only studies with high performance are more likely to be published. The risk of missing negative results may thus obscure the true variability of model performance, and it can mask the potential for overfitting. Such bias could also result in a misleading representation of a model's reliability when deployed in different clinical environments. For a robust assessment, it is essential that future research includes comprehensive reporting of both positive and negative findings and places greater emphasis on external validation to ensure that these high AUC values truly translate into clinical utility.

Finally, model generalizability remains a critical issue. The high reported AUC values might be indicative of models that perform well on internal test sets but fail to maintain their accuracy on external datasets that include varying imaging protocols, patient demographics, and clinical conditions. It is important to adopt multi-center studies and incorporate prospective validations to mitigate these risks. Only through rigorous testing on diverse, independent datasets can the true clinical performance of these deep learning models be ascertained, ensuring that the benefits of advanced image analysis and automation are reliably delivered in everyday clinical practice.

### Comparison of DL models with pathologist diagnoses

Recent studies have begun directly comparing the diagnostic performance of deep learning (DL) models with that of expert pathologists using statistical methods such as the DeLong test to evaluate differences in their receiver operating characteristic (ROC) curves. The DeLong test offers a robust method for comparing the areas under the curve (AUC) between DL models and human experts, thereby statistically determining whether the observed differences in diagnostic accuracy are significant. In several investigations, DL models have achieved AUCs comparable to or even exceeding those of pathologists. For example, in some studies, the DL models demonstrated AUCs near 0.97 on internal datasets, while the Delong test showed no statistically significant difference when compared with the performance of experienced pathologists. This suggests that the models are potentially reliable as diagnostic tools and may function effectively as second readers, offering valuable support in clinical decision-making (28, 29).

In addition to these direct comparisons, DL model performance has also been evaluated against established clinical gold standards, which include not only histopathological diagnoses but also relevant biomarkers (e.g., Ki-67 proliferation index) and patient outcomes such as recurrence rates and survival. By benchmarking against these gold standards, researchers have demonstrated that DL algorithms can replicate the diagnostic accuracy of traditional methods while providing faster, automated analyses that could reduce diagnostic turnaround times. However, while some studies report excellent agreement between DL model outputs and the gold standard, others highlight issues such as potential overfitting and reduced generalizability when models are applied to external datasets with different imaging protocols or patient demographics. These findings underscore the importance of comprehensive external validation and multi-institutional collaboration to ensure that AI tools are not only statistically robust (as confirmed by tests like the DeLong test) but also practically reliable in diverse clinical environments (30).

Together, these comparative analyses reinforce the potential role of deep learning as an adjunct diagnostic tool in clinical practice. They suggest that, when rigorously validated, DL models could enhance the accuracy and efficiency of meningioma grading, complementing the expertise of pathologists and contributing to more informed treatment planning. Nonetheless, ongoing research is needed to address challenges related to model overfitting and generalizability, ensuring that these advanced techniques maintain high performance across various clinical settings and patient populations (31).

### Implications for clinical practice

The high accuracy rates of these DL models suggest their potential applicability in clinical practice. For example, the 3DCNN model by Boaro et al. proved to be highly accurate, supporting its use as a reliable tool for histopathological grading (27). The study by Prakash et al. emphasized that such DL models could be integrated into computer-assisted diagnosis systems, thereby aiding radiologists in making more accurate and faster diagnoses. Mahmoud et al.'s findings further supported this, demonstrating that applying the VGG-19 model with AQO could significantly enhance diagnostic accuracy (22).

### Enhancing diagnostic efficiency

The incorporation of DL models in histopathological grading not only improves accuracy but also enhances the efficiency of the diagnostic process. Priya et al. noted that their proposed hybrid model could improve brain tumor detection, potentially reducing the workload of pathologists and radiologists (23). Similarly, Singh et al. highlighted that their HPS-GWO model can be used for initial screenings, helping to prioritize cases that require more detailed examination (24). Utilizing these models can lead to faster turnaround times for diagnoses, enabling quicker clinical decision-making and potentially enhancing patient outcomes.

This study has some limitations that can be addressed. Study heterogeneity was high, but this aligns with other machine learning meta-analyses and diagnostic meta-analyses. Not all articles specified whether the WHO 2016 classification of central nervous system tumors was used. We utilized the new Castle Ottawa checklist for the quality assessment of studies; however, more accurate assessment checklists designed explicitly for diagnostic studies could enhance the precision of quality assessment. Including only English papers could have contributed to the observed publication bias.

## Limitations

As this meta-analysis synthesizes findings from multiple peer-reviewed studies, we did not directly perform MRI preprocessing such as normalization or data augmentation. Instead, we extracted the reported preprocessing methodologies from each study. Among the included studies, several employed standard preprocessing techniques—such as z-score normalization, bias field correction, and various data augmentation methods—to mitigate inter-scanner variability and overfitting. However, we observed considerable heterogeneity in these methods, which may contribute to variability in reported performance metrics. In accordance with the STARD-AI guidelines, we have transparently documented these methodological details and acknowledge that the lack of uniform preprocessing across studies is a potential limitation of our analysis. Due to substantial methodological and clinical heterogeneity among the included studies—including variability in deep learning architectures, dataset characteristics, reported metrics, and preprocessing approaches—formal subgroup analyses or meta-regression were not feasible. We recognize this limitation and emphasize the need for future studies to adopt more standardized reporting practices, larger datasets, and consistent model evaluation metrics, thus enabling robust analyses of heterogeneity sources in future meta-analyses.

## Challenges and future directions

Despite the promising results, several challenges need to be addressed for the broader implementation of these deep learning models in clinical practice. These include the need for large, diverse datasets to train the models effectively and integrate these systems into existing clinical workflows. Ensuring the availability of high-quality, annotated datasets is crucial for training robust models. Moreover, the variability in histopathological images resulting from differences in staining techniques, imaging modalities, and patient demographics necessitates the use of diverse training datasets to improve model generalizability.

Another significant challenge is the interpretability of DL models. While these models achieve high accuracy, understanding the rationale behind their predictions remains a complex task. Developing methods to enhance the interpretability of deep learning (DL) models will be crucial for gaining the trust of clinicians and facilitating their adoption in clinical practice.

Integration into clinical workflows also poses a challenge. Implementing DL models in real-world settings requires seamless integration with existing medical imaging systems and electronic health records. Additionally, clinicians must be trained to utilize these models effectively and accurately interpret their results. Collaborative efforts between technologists, clinicians, and healthcare administrators will be crucial for overcoming these barriers.

Furthermore, to enhance the translational development of deep learning algorithms for medical imaging analysis, the efficacy of these systems must be tested by simulations of actual clinical tasks within the framework of a meticulously structured clinical trial. This will facilitate the selection and subsequent deployment of the most successful algorithms, which, while prioritizing patient privacy and safety and adhering to established certification and standardization protocols, can significantly enhance medical treatment and improve patient outcomes. The medical community must actively engage in this process, collaborating with computer scientists to direct development toward pertinent clinical inquiries and ensure the stringent validation of these algorithms (27).

Future research should address these challenges and validate the models in larger, multicenter studies to ensure their generalizability and robustness. Multicentric studies involving diverse patient populations and varying clinical settings will help assess the models' performance across different scenarios, ensuring their reliability and applicability in diverse clinical contexts. Furthermore, exploring the potential of transfer learning, where pre-trained models are fine-tuned on specific datasets, can help improve the models' performance with limited data.

The created deep learning segmentation models provide automatic and precise meningioma segmentation from MRI data. The DLM with automatic segmentation exhibited performance akin to that of the model utilizing manual segmentation. The automated segmentation method is likely to facilitate the application of radiomics in clinical practice and enhance its efficiency for the preoperative grading of meningiomas.

## Data Availability

The original contributions presented in the study are included in the article/supplementary material, further inquiries can be directed to the corresponding authors.
